# Antioxidant Activity and Inhibitory Effects of Black Rice Leaf on the Proliferation of Human Carcinoma Cells

**DOI:** 10.1155/2022/7270782

**Published:** 2022-06-11

**Authors:** Chorpaka Thepthanee, Chan-Chiung Liu, Hsu-Sheng Yu, Ho-Shin Huang, Chia-Hung Yen, Yen-Hsien Li, Maw-Rong Lee, Ean-Tun Liaw

**Affiliations:** ^1^Department of Food Science, National Pingtung University of Science and Technology, Pingtung 91201, Taiwan; ^2^R&D Center, King Herb BioMed, Tainan 71201, Taiwan; ^3^Department of Biological Science and Technology, National Pingtung University of Science and Technology, Pingtung 91201, Taiwan; ^4^Department of Chemistry, National Chung Hsing University, Taichung 420, Taiwan; ^5^Instrument Center, Office of Research and Development, National Chung Hsing University, Taichung 420, Taiwan

## Abstract

The leaves of black rice, well-known as postharvest agricultural waste, contain a rich source of antioxidants with multiple benefits for human health. In the present study, the ethyl acetate fraction obtained from black rice leaf was separated into five subfractions using Sephadex LH-20 column chromatography, and their antioxidant and anticancer activities were investigated. The results revealed that among all the subfractions, subfraction 5 (Sub5) showed the highest total phenolic and flavonoid values. The antioxidant activity was also superior in Sub5 (the IC_50_ values are 3.23, 31.95, and 72.74 *μ*g/mL, in the DPPH, ABTS, and reducing power assays, respectively) compared to the other subfractions. All subfractions, in a time-dependent manner, inhibited the proliferation of hepatoma (HepG2), breast (MCF-7), and colorectal (Caco-2) cancer cells, especially the Sub5. Thus, Sub5 was employed to conduct the cell cycle and cell apoptosis by flow cytometry. Sub5 significantly increased the accumulation of cells at the Sub-G1 phase in HepG2 cells (44.5%, at 48 h). Furthermore, it could trigger annexin V-detected apoptosis through mitochondrial and death receptor pathways accompanied by the suppression of PI3K/Akt and Erk signaling pathways. In addition, HPLC-DAD-MS/MS was conducted to characterize the bioactive constituents in the most potent antioxidant, cytotoxic, and apoptosis-inducing subfraction. Conclusively, Sub5 may have high potential as functional dietary supplements to inhibit the development of HepG2 liver cancer.

## 1. Introduction

Numerous aspects of modern lifestyles, such as insufficient nutrition, lack of physical activity, and an accumulation of pollutants (pesticides, heavy metals, food additives, and cigarette smoke), significantly increase the body's generation of free radicals to an alarming level [1], leading to changes in lipid and protein structure, inactivation of enzymes, aging of cells, and DNA damage, also known as oxidative stress [2]. Excessive oxidative stress can cause a wide range of health problems, including carcinogenesis, inflammation, aging, diabetes, cardiovascular disease, and others [3]. These symptoms can be treated or prevented by consuming antioxidant-rich edible plants, fruits, and vegetables, including phenolic acids and flavonoids [1].

Cancer is a complex and heterogeneous set of diseases that includes more than a hundred different diseases [4]. These diseases are characterized by out-of-control cell growth and spread of aberrant cells. Cancer became the second leading cause of human death throughout the world after cardiovascular diseases in 2020, causing more than 9.6 million deaths [5]. Lung, prostate, colorectal, stomach, and liver cancers are the prevalent forms of cancer in males, while breast, colorectal, lung, cervical, and thyroid cancers are the most common among females [5]. The common treatment strategies for cancer, such as radiotherapy, chemotherapy, drug treatment, surgical resection, or a combination of treatments, might cause some side effects for patients [6]. Therefore, the discovery of natural substances that have few side effects and can effectively inhibit the growth of cancer cells has become a priority [7].

Rice (*Oryza sativa* L.), a plant belonging to the Gramineae family, is the staple food for many people and an important agricultural commodity in many countries around the world, especially in Asia, revealing pharmacological properties that help prevent the formation of cancers [8]. Over the last decade, massive rice stems and leaves have been left in the fields after rice production, regarded as agricultural food waste. Usually, they are burned and have limited use as a fertilizer for the following crop. Rice grasses, both colored and non-colored, have been revealed extensively in recent years to have nutritional content, bioactive components, and pharmaceutical capacity [9, 10]. A previous study demonstrated that the extracts of Thai purple rice grass at the jointing stage contained significantly higher levels of phytochemicals and antioxidant ability than those of white rice grass [9]. Black glutinous rice grain sprouts (cv. BGR) were more effective in suppressing the proliferation of human T-lymphocyte (Jurkat), human liver (HepG2), and human colon (HCT116) cancerous cells than white rice sprouts (cv. RD6), due to their antioxidant ability and greater polyphenol and anthocyanin contents [10]. However, the identification of significant biological activity indicators continues to be a challenge. Thus, the aim of this work was to determine the antioxidant and anticancer effects of the phenolic fractions derived from black rice leaf using column chromatography. We also investigated induction of apoptosis through the two main apoptosis pathways, intrinsic and extrinsic, as well as suppression of the PI3K/Akt and Erk signaling pathways. Furthermore, the major bioactive compounds in the most potent anticancer fraction were identified using HPLC-DAD-MS/MS.

## 2. Materials and Methods

### 2.1. Extraction and Fractionation

Fresh black rice leaves were obtained from the Hualien District Agricultural Research and Extension Station Council of Agriculture, Hualien, Taiwan, in 2019. The sample was air-dried at 40 °C for 24 h until a constant weight was obtained and ground to powder using a stainless-steel blender (Long products industry & Trade Co., Ltd., Yongkang, China). The ground sample was extracted by adding 75% ethanol at a ratio of 1 : 10 (w/v) for 24 h. Then, the solution was filtered through filter paper (Whatman No. 1), and the entire extraction process was repeated twice on the residue collected from the previous filtration process. After evaporation of the ethanol solvent at 40 °C, the concentrated extract was lyophilized for 3 days.

The crude extract from black rice leaves was dissolved in distilled water and subsequently partitioned with hexane. After removing the hexane layer containing lipid, the aqueous fraction was partitioned with ethyl acetate to obtain the ethyl acetate fraction. It was filtered, concentrated at 40 °C, and then subfractionated through column chromatography [11]. The ethyl acetate fraction was dissolved in 95% (v/v) ethanol and then filtered through a 13 mm syringe filter (0.45 *μ*m, GHP membrane) before being subjected to a glass column (2.5 × 100 cm) packed with Sephadex LH-20 resin (Sigma-Aldrich, St. Louis, MO, USA) using 95% (v/v) ethanol as an eluent. The eluates were collected using a Bio-Rad Model 2110 fraction collector (Hercules, CA, USA), and the absorbance of each tube at 280 nm was recorded using an ELISA reader for the separation of ethyl acetate fraction. Collected fractions were grouped into five main fractions, evaporated, and lyophilized as mentioned above.

### 2.2. Total Phenolic Content (TPC) and Total Flavonoid Content (TFC)

The Folin–Ciocalteu method was employed to analyze the total phenolic content of ethanolic fractions of black rice leaves [12]. In the test tube, 100 *μ*L of ethanolic fractions were mixed with 2 mL of sodium carbonate (2%) and allowed to stand for 5 min. Then, 100 *μ*L of the Folin–Ciocalteu reagent (0.5 N) was added. The mixture was vortexed and incubated for 30 min in the dark. The absorbance of the mixture was recorded at 750 nm. The TPCs were determined as milligrams of gallic acid equivalents (GAE) per gram of dry weight (DW), and all measurements were conducted in triplicate.

The total flavonoid content of ethanolic fractions of black rice leaves was measured using the aluminum chloride colorimetric method [13]. In the test tube, 250 *μ*L of ethanolic fractions were diluted with 1.25 mL of distilled water and then mixed with 75 *μ*L of sodium nitrite (5%). After 6 min, 150 *μ*L of AlCl_3_ (10%) was added. The solution was incubated for 5 min in the dark before adding 0.5 mL of NaOH (1 M) and adjusting the volume to 2.5 mL with distilled water. The absorbance of the mixture was recorded at 510 nm. The TFCs were determined as milligrams of quercetin equivalents (QE) per gram of dry weight (DW), and all measurements were conducted in triplicate.

### 2.3. Antioxidant Activity

#### 2.3.1. DPPH Radical-Scavenging Activity

The capacity of the ethanolic fraction of black rice leaf to scavenge DPPH free radicals was conducted according to Hao et al.'s method [13]. In brief, 50 microliters of 0.1 mM DPPH methanolic solution was added to 200 *μ*L of BHA standard or the ethanolic fractions. The solutions were incubated for 30 min in the dark after mixing. The absorbance of samples was recorded at 517 nm. The results were measured as IC_50_, the concentration of the sample that inhibited DPPH radicals by 50%.

#### 2.3.2. ABTS^+^ Radical-Scavenging Activity

The capacity of the ethanolic fraction of black rice leaf to scavenge ABTS^+^ free radicals was conducted according to Hao et al.'s method [13]. ABTS^+^ (7 mM) solution was mixed with potassium persulphate (2.54 mM) overnight. After that, the mix was diluted with distilled water, and the absorbance was adjusted to 0.70 ± 0.02 at 734 nm. Twenty microliters of Trolox standard or the sample fractions were incubated with working solution of ABTS (180 *μ*L) at ambient temperature for 10 min in the dark, followed by a measurement at 734 nm. The results were measured as IC_50_, the concentration of the sample that inhibited ABTS radicals by 50%.

#### 2.3.3. Reducing Power Assay

The reducing power assay was conducted according to Baek et al.'s method [14] with minor modifications. Briefly, 500 *μ*L of BHA standard or each sample fractions were mixed with 500 *μ*L of buffer solution (0.2 M, pH 6.6 phosphate buffer combined with 1% potassium ferricyanide solution) and then hatched in a water bath at 50 °C for 20 min and cooled immediately on ice. Trichloroacetic acid (10%, 500 *μ*L) was added to the mix solution. After centrifugation at 2500 g for 10 min, the supernatant (100 *μ*L) was diluted with an equal volume of distilled water; then, ferric chloride solution (0.1%, 20 *μ*L) was added. The absorbance of the resulting solution was recorded at 700 nm. The data were measured as the IC_50_ value, the concentration of the sample that exhibited absorption of 0.5.

#### 2.3.4. Cell Line and Cell Culture

The Bioresource Collection and Research Center (BCRC, Hsinchu, Taiwan) provided human liver cancer cell line HepG2, human breast cancer cell line MCF-7, human colon cancer cell line (Caco-2), and normal mouse liver cell line FL83B. HepG2 and Caco-2 cells were cultured in DMEM supplementary with 10% FBS. MCF-7 was maintained in MEM with 10% FBS. FL83B was cultured in F12K medium with 10% FBS. All cells were incubated at 37 °C in a 5% CO_2_ incubator.

### 2.4. Antiproliferative Assay

Cancer and normal cells (1 × 10^4^ cells/well) were seeded on 96-well plates and incubated overnight. Then, 100 *μ*L per well of various concentrations of the sample (0, 25, 50, 100, 200, and 400 *μ*g/mL) was added and incubated for 24, 48, and 72 h. After incubation with 20 *μ*L of MTT solution (5 mg/mL) for 4 h, the medium was replaced with 100 *μ*L of DMSO to dissolve the violet crystals of formazan, and the plates were shaken on a microplate shaker (100 rpm) for 30 min in the dark, followed by a measurement at 570 nm. 5-Fluorouracil (5-FU), a chemotherapy drug, was used as a positive control.

### 2.5. Cell Cycle Assay

Briefly, HepG2 cells at a density of 3 × 10^5^ cells/well were added into 6-well plates. After incubation for 24 h, the cells were treated with different concentrations of subfraction 5 (0, 50, 100, 200, and 400 *μ*g/mL) and 5-FU (25 *μ*g/mL) for 24 and 48 h. The cells were harvested by trypsinization and then washed with cold PBS three times by centrifugation at 3000 rpm for 5 min. After cell fixation with ice-cold 70% ethanol overnight, the cells were washed with PBS and labeled with propidium iodide (PI) staining solution for 30 min in the dark. Cell cycle arrest and sub-G1 accumulation were analyzed using flow cytometry (BD FACS Canto II, USA).

### 2.6. Apoptosis Assay

HepG2 cells (3 × 10^5^ cells/well) were added into 6-well plates overnight and incubated with subfraction 5 (0, 50, 100, 200, and 400 *μ*g/mL) for 24 and 48 h. Harvested cells were double labelled with FITC conjugated annexin V and PI for 15 min in a dark environment; then, the populations of live, early apoptotic, late apoptotic, and necrotic cells were analyzed using flow cytometry (BD FACS Canto II, USA).

### 2.7. Western Blot Analysis

HepG2 cells were seeded into a 10^2^ cm dish at a density of 1 × 10^6^ cells/dish and incubated with various concentrations of subfraction 5 (0, 50, 100, 200, and 400 *μ*g/mL) and 5-FU (25 *μ*g/mL) for 24 h. The cells were harvested by cell scraping, rinsed with ice-cold PBS twice, and centrifuged at 3,000 rpm for 5 min. Pellet cells were lysed in a cold modified RIPA buffer with a protease inhibitor cocktail and then vortexed for 20 min at 4 °C. After centrifugation at 12,000 rpm for 20 min at 4 °C, the supernatant was collected to measure the total protein concentration using the Coomassie Plus (Bradford) Assay Kit (Thermo Science, Rockford, IL, USA). The protein samples were isolated by 10% SDS-PAGE and transferred to polyvinylidene difluoride membranes by electroblotting. Next, they were blocked with 5% bovine serum protein (BSA) in TRIS-buffered saline with Tween 20 (TBST) for 1 h and soaked in primary antibodies (1 : 1000 dilutions) overnight in a 4 °C microplate shaker. The membranes were washed with TBST three times subsequently dipped in secondary antibodies for 1 h, and washed again with TBST three times. Finally, they were visualized with ECL solution (GE Health Care, Buckinghamshire, UK), and the protein levels were analyzed using Image J software (National Institutes of Health, USA).

### 2.8. Identification of Phenolic Compounds

The phenolic acid and flavonoid components and contents in subfraction 5 were identified using high-performance liquid chromatography coupled to diode array detection (HPLC–DAD) and electrospray ionization tandem mass spectrometry (ESI-MS/MS). In brief, 1 mL, 40 *μ*g/L of Sub5 was mixed with the equal volume of internal standard, catechin (40 *μ*g/L). The mixing solution was filtered through 0.45 *μ*m membrane filters before analysis. The sample was analyzed using a Thermo Scientific Dionex Ultimate 3000 HPLC system (Thermo Fisher, San Jose, CA, USA) that consisted of a binary pump, a diode array detector, and an autosampler. A C18 (Thermo ScientificTM HypersilTM BDS C18, 250 mm × 4.6 mm, 5 *μ*m particles) column was employed for chromatographic separation of the sample at room temperature. Mobile phase A was distilled water containing 0.5% acetic acid, whereas mobile phase B was 100% acetonitrile. The gradient elution was performed according to our previous work [12]. During the elution process, the flow rate was set at 0.5 ml/min. The injection volume was 5 *μ*L, and the sample's phytochemical components were identified at 280 nm. Under mass spectrometry conditions, the ESI negative ion mode was used for detection in full-scan mode using the following parameters: sheath gas flow rate, 50 arbitrary units; aux gas flow rate, 20 arbitrary units; sweep gas flow, rate 2 arbitrary units; spray voltage, 4.5 kV; capillary temperature, 270 °C; S-lens RF level 55.0; and mass range, m/z 100–1200. Full scan mode data-dependent MS/MS were acquired using data-dependent Top5 (ddMS2/Top5) with a mass resolution of 70,000. The settings for the full scan and the ddMS2-top5 scan were established as previously described [12].

The calibration curve for 25–125 *μ*g/L of luteolin (y = 9336.5x + 13431; *R*^2^ = 0.9954) was used to quantify the compound. Data are expressed as milligrams of luteolin equivalents per gram (mg/g).

### 2.9. Statistical Analysis

All the measurements were conducted in triplicate using SPSS 24.0 (SPSS Inc., Chicago, IL, USA) to carry out statistical analysis (means ± standard deviations). The significant intergroup differences were compared by one-way ANOVA with Duncan's test and set at *p* < 0.05. The *t*-test was calculated the same as the prior calculation for two-group comparisons (^∗^*p* < 0.05 and ^∗∗^*p* < 0.01).

## 3. Results and Discussion

### 3.1. Extraction Yields, TPC, and TFC of Ethyl Acetate Subfractions Derived from Black Rice Leaf

**Figure 1:**
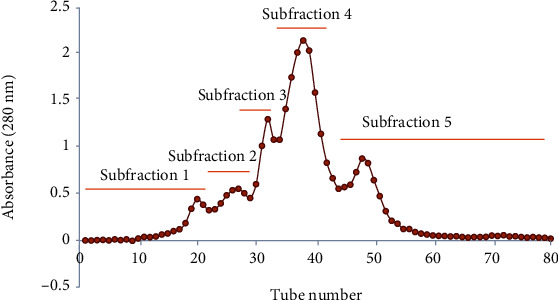
Sephadex LH-20 column chromatogram of ethyl acetate subfraction from black rice leaf ethanolic extract.

Among all fractions, ethyl acetate fraction (EtOAc) possessed the strongest anti-HepG2 liver carcinoma cells activity, with the presence of several phenolic constitutes [12]. Thus, this present study was performed on the ethyl acetate fraction to isolate the anticancer compounds. The EtOAc was further isolated on a glass column (2.5 × 100 cm) packed with Sephadex LH-20 resin. Sephadex® LH-20 is a liquid chromatographic medium commonly utilized in molecular size exclusion of various natural compounds [15]. Numerous studies have been conducted on the fractionation and purification of phenolic compounds using Sephadex LH-20 [12, 16, 17]. The chromatogram for EtOAc fraction loaded on a Sephadex LH-20 column is shown in Figure 1. The various fractions collected following fractionation were measured at a wavelength of 280 nm to detect phenolic substances in general. Five main subfractions (Sub 1–5) were obtained from the ethyl acetate fraction (Figure 1). The yields of subfraction 1 (collection tubes 9–23), subfraction 2 (collection tubes 24–29), subfraction 3 (collection tubes 30–34), subfraction 4 (collection tubes 35–44), and subfraction 5 (collection tubes 45–70) were 15.05, 13.93, 18.19, 31.49, and 12.88% (w/w, on a dry weight basis), respectively (Table 1). The order of extraction yields was as follows: Sub4 > Sub3 > Sub1 ≥ Sub2 ≥ Sub5 (*p* < 0.05). The Sub4 demonstrated the highest yield (31.49%), approximately 2.44 times greater than the minimum extract yield (12.88%) obtained with Sub5.

The values of TPC and TFC in the ethyl acetate subfraction 1–5 of black rice leaf are exhibited in Table 1. The TPC and TFC contents of Sub1–5 were significantly different (*p* < 0.05), in the range of 101–631.36 mg GAE/g DW and 359.83–2117.33 mg QE/g DW, respectively. When comparing all subfractions, the highest contents of TPC and TFC were observed in Sub5, whereas the lowest values of both compounds were found in Sub1. These results demonstrate that the most active natural compound could be found in Sub5. Phenolic acids and flavonoids have long been recognized as powerful antioxidants and anticancer agents [18–20].

### 3.2. Antioxidant Activities of Ethyl Acetate Subfractions Derived from Black Rice Leaf

The antioxidant activity of subfractions 1–5, which mainly contained different phenolic compounds, was determined using free radical-scavenging and reducing power assays. The results are expressed as IC_50_ values.

As shown in Table 2, subfraction 5 presented the strongest DPPH and ABTS radical-scavenging activity (IC_50_, 3.23 ± 0.05 *μ*g/mL and 31.95 ± 1.51 *μ*g/mL, respectively). However, the weakest DPPH and ABTS radical-scavenging activity was found in subfraction 1, which may be due to the low amounts of TPC and TFC in this fraction.

The reducing power of the subfractions derived from black rice leaf was represented as the concentration of the sample providing an absorbance of 0.5 (IC_50_ values), as shown in Table 2. The IC_50_ values varied from 72.74 ± 0.71 *μ*g/mL to 1511.11 ± 9.75 *μ*g/mL, and the reducing power decreased in the following order: Sub5 > Sub3 > Sub2 > Sub4 > Sub1 (*p* < 0.05). Significant differences in the reducing power of the distinct fractions might be explained by the variances in their chemical compositions. Additionally, Sub5 illustrated the highest reducing power, consistent with the scavenging ability of both DPPH and ABTS radicals. Surprisingly, Sub5 demonstrated the same or greater antioxidant capacity when compared to synthetic antioxidants, i.e., BHA, Trolox, and ascorbic acid. Thus, Sub5 seemed to have a high potential as a source of natural antioxidants for reducing oxidative damage and providing health protection in the human body.

Phytochemicals in Sub5 fraction collected and concentrated with column chromatography on Sephadex LH-20 showed a positive association with antioxidant activity. Thus, Sub5 was chosen for further study of its anticancer activity.

### 3.3. Antiproliferative Activity of Ethyl Acetate Subfractions Derived from Black Rice Leaf against Several Human Cancer Cell Lines

The antiproliferative activities of the five subfractions against three human cancer cells for 24, 48, and 72 h were evaluated by an MTT assay, and the results are presented in Table 3. Among the five phenolic subfractions, the Sub5 exhibited the most remarkable capacity against HepG2, MCF-7, and Caco-2, with average half-maximal inhibitory concentration (IC_50_) values of 74.42, 95.33, and 82.19 *μ*g/mL, respectively, for 72 h. The results indicated that active compounds were mainly concentrated in subfraction 5. According to the US National Cancer Institute (NCI) [21], the Sub5 exhibited moderate cytotoxicity (IC_50_ ranged from 21 to 200 *μ*g/mL), whereas fluorouracil (5-FU) had high cytotoxicity (IC_50_ ≤ 4 *μ*g/mL) against HepG2 and Caco-2 cells with IC_50_ values of 2.41 ± 0.08 and 2.05 ± 0.01 *μ*g/mL, respectively. In contrast, the subfractions did not significantly affect the cell proliferation of normal mouse liver cells FL83B with the highest concentration tested (Figure S1), demonstrating that the toxicity of the samples was limited to cancerous cells. Thus, this study confirmed that Sub5, a phenolic-rich fraction, not only contains more antioxidant compounds but demonstrates better anticancer activity. Furthermore, positive correlations between phytochemical content and antiproliferative activity were found in the current investigation in which the *r*-values ranged from 0.590 to 0.980 (Table S1). According to previous studies, polyphenol can block the growth and proliferation of many forms of cancer [19, 20]. Nevertheless, no significantly correlative relationships were established between antioxidant and antiproliferative properties against HepG2 liver cancer cells (Table S1). This might be attributed to the structural specificity of the anticarcinogenic compounds [22].

### 3.4. Morphological Changes in HepG2 Cells following Treatment with Sub5

**Figure 2:**
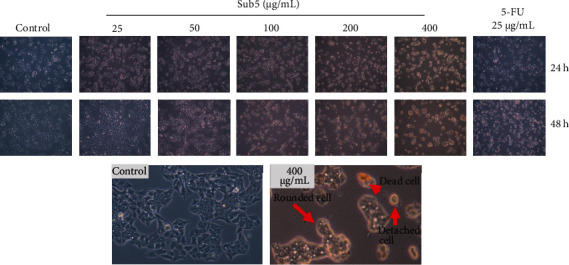
Effect of subfraction 5 on the cell morphology change in HepG2 cells. The cells were imaged by ZEISS Primovert microscope (100X).

As presented in Figure 2, the subfraction 5 treatment resulted in morphological alterations in human liver cancer cells. The apoptotic features of HepG2 cells after incubation with Sub5, included cell body shrinkage, membrane blebbing, nuclear fragmentation, and the creation of apoptotic bodies, were detected by a light microscope. HepG2 cells exposed to Sub5 (50–400 *μ*g/mL) for 24 and 48 h exhibited an increase in the population of apoptotic cells and a reduction in the population of living cells. Moreover, the cells were detached and suspended in the medium after being treated with Sub5. At the maximum dose of 400 *μ*g/mL, the rounded cell morphology and a reduction in cell population were observed, as well as a decrease in cell adhesion. This result suggests that apoptosis plays a crucial role in the inhibition of cancer cell proliferation.

### 3.5. Sub5 Triggers the Accumulation in Sub-G1 on HepG2 Cells

**Figure 3:**
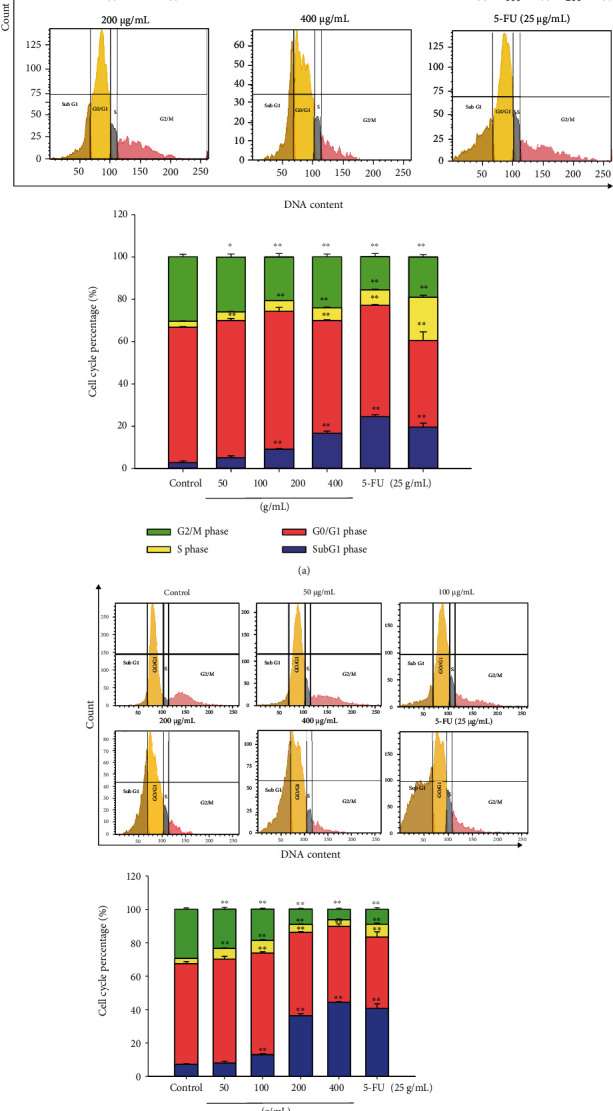
The effect of subfraction 5 (Sub5) induced the number of cells in Sub-G1 on HepG2 cells. Propidium iodide (PI) staining with flow cytometry was used to determine the cell-cycle distributions. The cells were treated with Sub5 (0 to 400 *μ*g/mL) or 5-FU (25 *μ*g/mL) for 24 h (a) and 48 h (b). The percentage of the cell-cycle phases are displayed in a bar chart. ^∗^ and ^∗∗^ represent significantly different results at *p* < 0.05 and *p* < 0.01, respectively, from the untreated cells.

The detection of the cell-cycle distribution by quantitation of DNA content was carried out with PI staining using flow cytometry. After 24 and 48 h treatments, the cell cycle was evaluated for various concentrations (50–400 *μ*g/mL) of subfraction 5. As shown in Figure 3, Sub5 significantly induced the number of cells at the Sub-G1 phase in HepG2 cells depending on the dose and time, which implied apoptotic cell death. When the cells were treated with Sub5 for 24 and 48 h, the rate of cells in the Sub-G1 phase increased from 5.13 to 24.43% and from 8 to 44.5%, respectively, compared with the untreated cells. These results might be due to Sub5 exhibiting the highest phytochemical contents (TPC and TFC). A previous study showed that flavonoid-rich *M. modestum* leaf methanolic extract induced cell cycle arrest at the Sub-G1 phase in human myeloid leukemia (RAJI) cells, indicating an increase in the population of apoptotic cells [23].

### 3.6. Sub5 Triggers Apoptosis in HepG2 Cells

**Figure 4:**
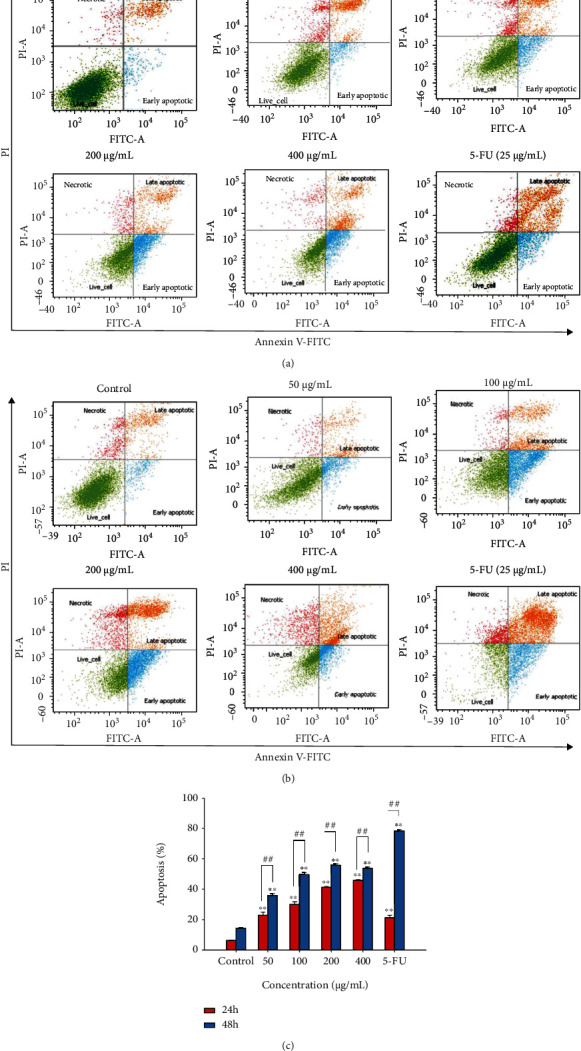
The effect of subfraction 5 (Sub5) on HepG2 cell apoptosis. Cell apoptosis was measured using annexin V-FITC/PI staining with flow cytometry. HepG2 cells were treated with Sub5 (0 to 400 *μ*g/mL) or 5-FU (25 *μ*g/mL) for 24 h (a) and 48 h (b). The percentage of apoptosis was estimated using the sum of the populations of annexin V-FITC (+) and PI (+/−) (c). ^∗∗^ and ^##^ represent significantly different results at *p* <0.01 from the untreated cells and treatment time, respectively.

In order to confirm that the apoptotic cell death accumulated in the Sub-G1 phase was triggered by Sub5 in hepatocellular carcinoma cells, we conducted an annexin V/PI staining assay using flow cytometry. Subfraction 5 effectively lowered the number of viable cells and enhanced apoptotic cells in a concentration- and time-dependent manner as presented in Figure 4. The percentage of total apoptotic cells (early apoptosis plus late apoptosis) was significantly increased from 11.63% to 45.83% and from 13.97% to 55.73% with the increase in concentrations of Sub5 for 24 and 48 h, respectively. We also found that the proportion of apoptotic cells rose in both early and late apoptosis. These results demonstrated the potential of subfraction 5 to trigger apoptosis in HepG2 cells, possibly due to its higher phenolic content and greater antioxidant capacity. A previous study also reported that black rice bran induced apoptosis in hepatocellular carcinoma cells [24], which is in agreement with our study. Numerous research has demonstrated that plant polyphenols contribute to antioxidant, antiproliferative, and apoptosis in several cancer cell lines [19, 20].

### 3.7. Sub5 Activates Intrinsic and Extrinsic Apoptotic Pathways in HepG2 Cells

**Figure 5:**
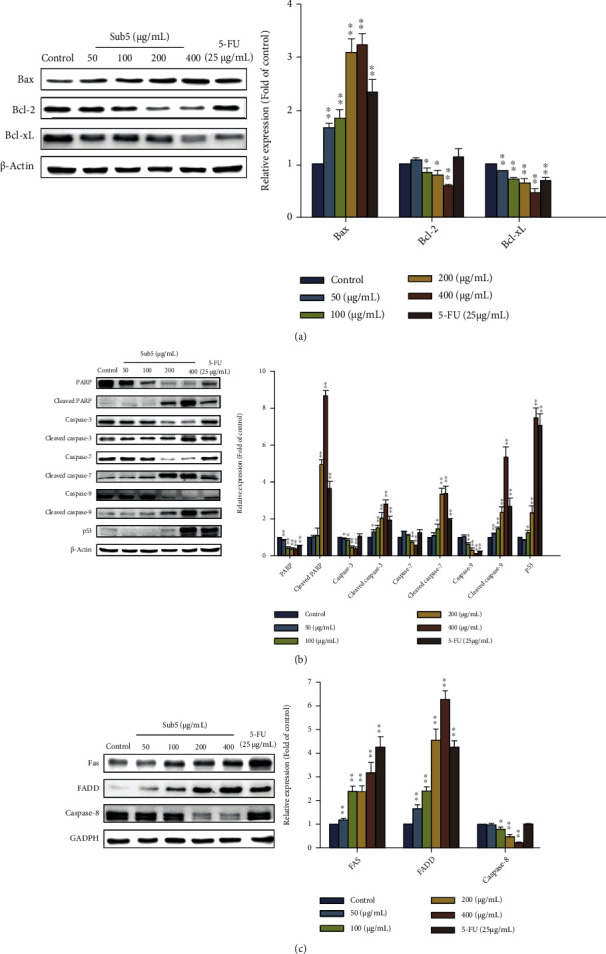
Effect of Sub5 on the expression of Bcl-2 family proteins (a), intrinsic apoptotic proteins (b), and extrinsic apoptotic proteins (c) in HepG2 cells. The cells were incubated with subfraction 5 (24 h, 0 to 400 *μ*g/mL) or fluorouracil (5-FU) (25 *μ*g/mL), and the levels of protein expression were investigated by immunoblot analysis. ^∗^ and ^∗∗^ represent significantly different results at *p* < 0.05 and *p* < 0.01, respectively, from the untreated cells.

To confirm whether the subfraction 5 could indeed induce apoptosis, the next objective was to investigate the effects of Sub5 on the expression of key proteins in the intrinsic and extrinsic apoptosis pathways using Western blot analysis. The mitochondrion-mediated apoptosis pathway begins with an increase in the ability of mitochondria to release apoptogenic factors such as cytochrome *c* into the cytoplasm caused by the regulation of Bcl-2 family proteins, thereby cells [25]. In HepG2 cells treated with Sub5 (50–400 *μ*g/mL) for 24 h, the levels of pro-apoptotic protein (Bax) were significantly increased compared to the control group, whereas the expression of anti-apoptotic proteins (Bcl-2 and Bcl-xL) markedly downregulated in a concentration-dependent manner (Figure 5(a)). As these results, it enhanced the cleavage and activation of an initiator caspase (caspase-9), an effector caspase (caspase-3 and -7), and poly (ADP-ribose) polymerase (PARP) protein (Figure 5(b)). p53, a tumor suppressor protein, plays a crucial role in preventing tumorigenesis through inducing apoptosis via multiple pathways, including cell cycle arrest [26]. As presented in Figure 5(b), the Sub5 significantly upregulated the expression of p53 compared to the control group. In addition, the expression of key proteins in the pathway mediated by extrinsic death receptors was also determined. The expression of Fas and FADD was dramatically increased in human liver cancer cells after Sub5 treatment for 24 h, while caspase-8 levels were also significantly decreased in a concentration-dependent manner (Figure 5(c)). These results illustrate that Sub5 triggered HepG2 apoptosis was involved in the intrinsic mitochondrial pathway and extrinsic death receptor pathway.

### 3.8. Sub5 Inhibits PI3K/Akt and Erk Signaling Pathway in HepG2 Cells

**Figure 6:**
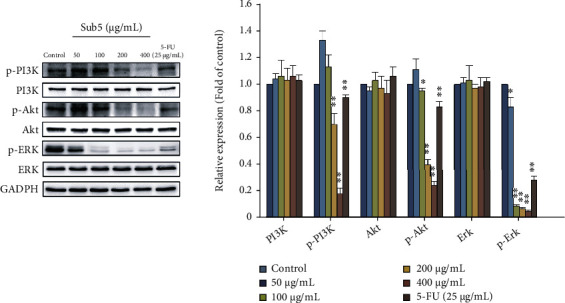
Effect of Sub5 on the activation of PI3K, Akt, and Erk. HepG2 cells were incubated with subfraction 5 (24 h, 0 to 400 *μ*g/mL) or fluorouracil (5-FU) (25 *μ*g/mL), and the levels of protein expression were investigated by immunoblot analysis. ^∗^ and ^∗∗^ represent significantly different results at *p* < 0.05 and *p* < 0.01, respectively, from the untreated cells.

In HepG2 cells treated with subfraction 5 for 24 h, we explored the suppression mechanisms on the PI3K/Akt and Erk signaling pathway by Western blotting. The PI3K/Akt pathway plays an essential role in carcinogenesis by controlling the growth and death of tumor cells [27]. Compared with the untreated group, Sub5 (200 and 400 *μ*g/mL) dramatically reduced the amount of PI3K and Akt protein phosphorylation, with no significant differences in the total PI3K and Akt expression levels in HepG2 (Figure 6). A previous study revealed that the activated phosphorylated Akt interacting with downstream target proteins regulated various functions of cell processes, including cell survival, growth, and proliferation, through controlling apoptosis by inhibiting proapoptotic proteins (Bad, Bax, and Bim) and caspase-9 [28]. The Erk signaling pathway involves enhancing either intrinsic or extrinsic apoptotic pathways through inducing the release of mitochondrial cytochrome c or activation of caspase-8 [29]. Figure 6 also shows that the Sub5-treated HepG2 cells exhibited downregulation of the p-Erk protein in a dose-dependent manner, compared with the untreated group, whereas the expression of Erk remained stable. Numerous polyphenol-rich herbs, notably Chinese bayberry leaves, possess anticancer properties through blocking this pathway's activity [30]. Additionally, flavonoids such as orientin and luteolin cause apoptosis via the Erk pathway in a number of cancer cell types [31, 32]. Collectively, these data suggest that Sub5 suppresses cell proliferation and promotes apoptosis via the PI3K/Akt and Erk signaling pathways in liver cancer cells (HepG2).

### 3.9. HPLC-DAD-MS/MS

**Figure 7:**
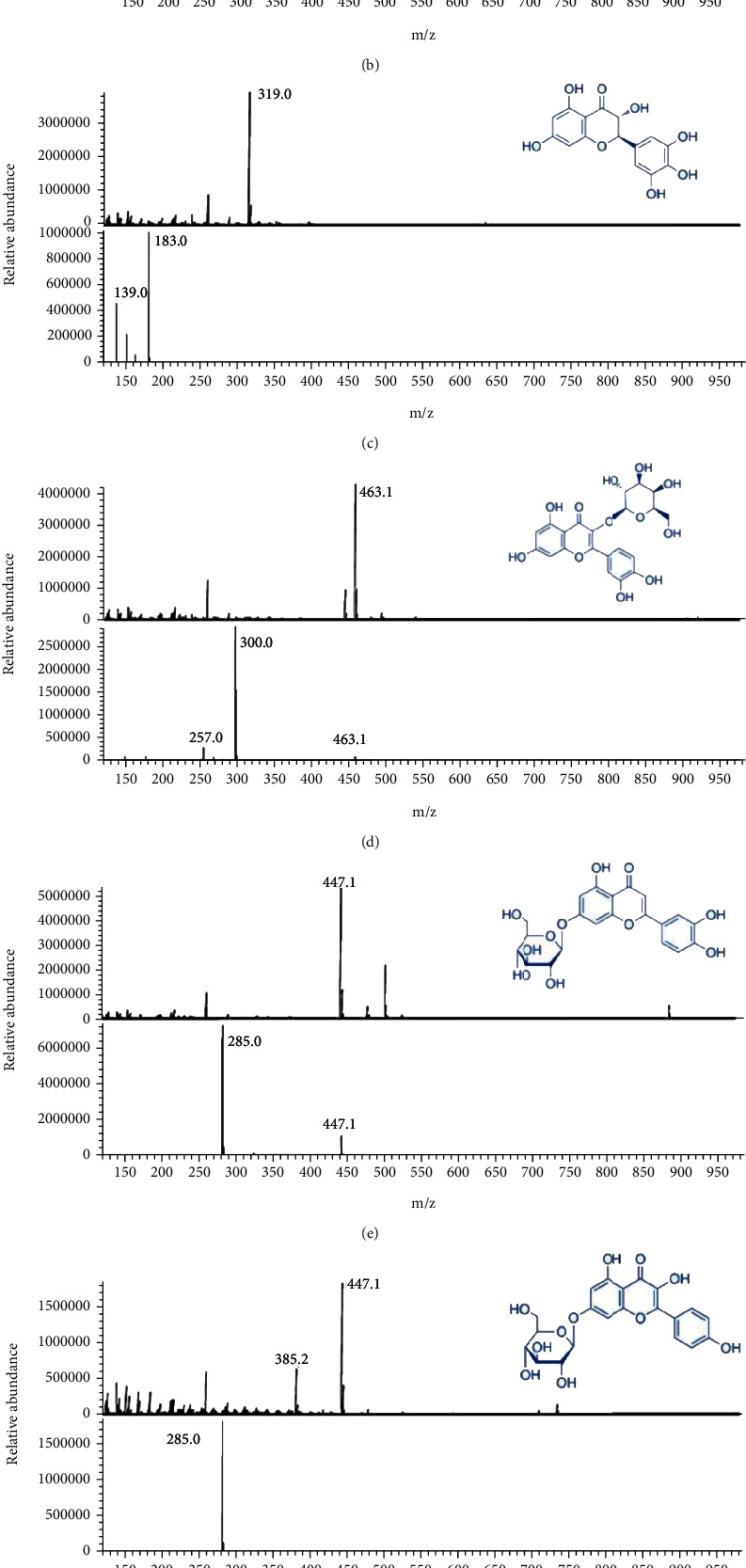
Total ion chromatogram of HPLC-DAD-MS/MS with UV detection at 280 nm from subfraction 5 mixed with 20 *μ*g/mL catechin in negative electrospray ionization mode (a). Full scan mass spectra and product ion mass spectra of [M-H]^−^ ions of peak 1 (b), peak 2 (c), peak 3 (d), peak 4 (e), peak 5 (f), and peak 6 (g). The peaks are marked with the names of the substances mentioned in Table 4.

In this study, phenolic compositions of subfraction 5 were identified by HPLC-DAD-MS/MS, since this fraction exhibited the greatest TPC and TFC values, as well as showed the best antioxidant and anticancer activity. According to our data, there were six phenolic compounds, luteolin-8-C-glucoside, 3,3,4,5,5,7 hexahydroxyflavanone, quercetin-3-galactoside, luteolin-7-O-glucoside, kaempferol-7-O-glucoside, and luteolin, present in subfraction 5 after subfractionation of the ethyl acetate fraction (Figure 7, Table 4). Remarkably, luteolin (peak 6) was the most abundant component in Sub5; the mass spectrum exhibited a molecular ion peak at m/z 285 and formed product ions at m/z 65, 107, 133, 151, 175, 199, 217, and 285 in the negative ion mode (Figure 7). This was matched to the MS/MS fragmentation pattern of a commercial standard, confirming the presence of luteolin in subfraction 5. The second highest substance was luteolin glycosides, luteolin-8-C-glucoside (peak 1), and luteolin-7-O-glucoside (peak 4). Luteolin and its glycosides are found in many plants and have a wide range of pharmacological properties including antioxidant, anticancer, anti-inflammatory, antidiabetic, and antibacterial activity [31, 32]. They can suppress the growth and proliferation of various cancer cells, such as gastric, prostate, liver, and colon, via a variety of pathways including inhibiting the progression of cell cycle, triggering apoptosis cell death, affecting the cell kinase pathway, blocking transcription factors, and controlling cellular oxidation and reduction reactions [33]. A previous study revealed that luteolin, derived from *Ixeris sonchifolia* Hance, inhibited the proliferation of HepG2 liver cancer cells by arresting the cell cycle on the G1 phase [34]. Furthermore, luteolin has been found to be a safe and effective chemopreventive agent against malignant tumors *in vivo* [33]. The majority of luteolin glycosides are 7-O-glycoside; they can also be found as 8-C-glycoside (orientin), which are more soluble and stable than aglycone [35]. However, luteolin aglycone exhibits a stronger anti-inflammatory activity than luteolin-7-O-glycoside in LPS-activated RAW 264.7 macrophage cells [36]. Additionally, another study demonstrated that the free form of luteolin exhibited a better antidiabetic effect than luteolin-7-O-glycoside luteolin [37]. Another study demonstrated that the luteolin-8-C-glycoside from flax straw inhibited the proliferation of breast cancer cells (MCF-7), which may be attributed to the control of Bcl-2 apoptosis-related gene expression as well as the triggering of apoptosis through the caspase-dependent pathway [38]. It is interesting to note that luteolin, luteolin-8-C-glucoside, and luteolin-7-O-glucoside are strongly associated with the antioxidant and anticancer capacities of black rice leaf [36–38]. Further exploration through *in vivo* experiments using animal models, as well as clinical trials, are required to obtain a greater understanding of the molecular mechanisms of and the development of proper bioactive substances from black rice leaf.

## 4. Conclusions

The extraction and fractionation methods were employed to identify the primary components in black rice leaf, highlighting the most important for their antioxidant and anticancer ability. Subfraction 5 possessed the highest phytochemical contents (TPC and TFC) and antioxidant capabilities (DPPH, ABTS, and reducing power), suggesting a strong potential for use as a natural antioxidant. Our study also revealed that subfraction 5 had the greatest cytotoxicity against human cancer cell lines. It suppressed the proliferation of HepG2 cells by arresting the cell cycle at the Sub-G1 phase and triggering apoptotic cell death. Furthermore, subfraction 5 had the ability to activate both the intrinsic and extrinsic apoptosis pathways and to inhibit the survival pathways, such as the PI3K/Akt and Erk signaling pathways, in hepatocellular carcinoma cells. The HPLC-DAD-MS/MS analysis demonstrated that the main compound in subfraction 5 derived from black rice leaf was luteolin: luteolin-8-C-glucoside and luteolin-7-O-glucoside. To summarize, subfraction 5 is not only a source of potential phytochemicals as an antioxidant and anticancer agent but also might be developed for use in functional foods and pharmaceutical applications.

## Figures and Tables

**Table 1:** Yields, TPC, and TFC of various subfractions derived from black rice leaf.

Subfraction	Yields (%)	Total phenolic content (mg GAE/g DW)	Total flavonoid content (mg QE/g DW)
Subfraction 1 (Sub1)	15.05 ± 0.67^cd^	101.00 ± 3.15^e^	359.73 ± 3.15^e^
Subfraction 2 (Sub2)	13.93 ± 0.45^c^	375.61 ± 3.15^d^	428.40 ± 8.72^d^
Subfraction 3 (Sub3)	18.19 ± 0.83^b^	500.15 ± 12.87^c^	677.33 ± 11.55^c^
Subfraction 4 (Sub4)	31.49 ± 0.79^a^	526.82 ± 8.08^b^	737.33 ± 30.55^b^
Subfraction 5 (Sub5)	12.88 ± 0.86^d^	631.36 ± 8.95^a^	2117.33 ± 23.09^a^

GAE: gallic acid equivalent and QE: quercetin acid equivalent. The results of phenolic and flavonoid contents are expressed as mean ± SD (*n* = 3). The letters ^a-e^ in the same row represent significant differences (*p* < 0.05).

**Table 2:** Antioxidant activities of various subfractions derived from black rice leaf.

Subfraction	IC_50_ (*μ*g/mL)		
	DPPH^•^	ABTS^•+^	Reducing power
Subfraction 1 (Sub1)	157.91 ± 3.14^a^	700.45 ± 16.92^a^	1511.11 ± 9.75^a^
Subfraction 2 (Sub2)	33.96 ± 0.47^b^	121.89 ± 5.31^b^	392.79 ± 19.70^c^
Subfraction 3 (Sub3)	30.19 ± 0.73^c^	70.47 ± 0.56^c^	261.81 ± 7.69^d^
Subfraction 4 (Sub4)	35.22 ± 0.79^b^	109.98 ± 2.73^b^	737.33 ± 30.55^b^
Subfraction 5 (Sub5)	3.23 ± 0.05^d^	31.95 ± 1.51^e^	72.74 ± 0.71^e^
BHA	4.46 ± 0.06^d^	—	—
Trolox	—	52.04 ± 0.10^d^	—
Ascorbic acid	—	—	49.63 ± 0.66^e^

The values of IC_50_ are displayed as mean ± SD (n =3). The letters ^a-e^ in the same row represent significant differences (*p* <0.05).

**Table 3:** Antiproliferative activity of ethyl acetate subfractions derived from black rice leaf against several human cancer cell lines.

Cell lines	IC_50_ (*μ*g/mL)					
	Sub1	Sub2	Sub3	Sub4	Sub5	5-FU
HepG2						
24 h	>400	>400	>400	>400	255 ± 3.25^a^	177.73 ± 3.77^b^
48 h	385.40 ± 3.39^a^	>400	>400	236.79 ± 2.34^b^	132.17 ± 12.54^c^	2.50 ± 0.01^d^
72 h	369.15 ± 2.35^a^	>400	310.31 ± 5.06^b^	101.51 ± 9.36^c^	74.42 ± 0.94^d^	2.41 ± 0.08^e^
MCF-7						
24 h	>400	>400	396.34 ± 13.79^a^	>400	233.34 ± 19.24^c^	298.23 ± 0.83^b^
48 h	>400	>400	290.55 ± 1.94^a^	192.39 ± 9.27^b^	174.13 ± 5.65^d^	184.05 ± 4.28^c^
72 h	338.68 ± 16.54^a^	283.78 ± 4.87^b^	172.78 ± 1.53^c^	160.80 ± 0.30^d^	95.33 ± 2.96^e^	37.90 ± 2.60^f^
Caco-2						
24 h	>400	>400	>400	>400	334.32 ± 7.38^a^	2.69 ± 0.03^b^
48 h	>400	>400	372.60 ± 8.73^a^	>400	179.71 ± 2.03^b^	2.06 ± 0.02^c^
72 h	>400	>400	291.02 ± 1.17^b^	308.28 ± 4.49^a^	82.19 ± 2.75^c^	2.05 ± 0.01^d^

IC_50_: concentration of the sample with 50% inhibition of cancer cells. The values of IC_50_ are displayed as mean ± SD (*n* = 3). 5-FU, fluorouracil, is the reference drug. The letters ^a-e^ in the same row represent significant differences (*p* < 0.05).

**Table 4:** The retention time and mass spectral characteristics of the bioactive compounds detected in subfraction 5 by HPLC-DAD-MS/MS.

Peak	RT (min)	Tentative identification	[M-H]^−^ *(m/z)*	MS/MS *(m/z)*	Amount (mg/g)	Ref.
1	46.77	Luteolin-8-C-glucoside	447	297, 327, 357	55.86 ± 0.06	[39]
2	50.09	3,3,4,5,5,7 Hexahydroxyflavanone	319	139, **183**	10.04 ± 0.08	[40]
3	58.25	Quercetin-3-galactoside	463	257, **300**, 463	28.59 ± 0.26	[41]
4	59.25	Luteolin-7-O-glucoside	447	**285**, 447	55.45 ± 0.60	[41]
5	70.85	Kaempferol-7-O-glucoside	447	**285**	12.41 ± 0.25	[42]
6	90.39	Luteolin	285	65, 107, **133**, 151, 175, 199, 217, 285	107.41 ± 5.53	Standard

RT: retention time and Ref.: reference. The most abundant fragments are marked in bold.

## Data Availability

All data generated or analyzed during this study are included in the published article.

## Supplementary Materials

Supplementary Materials The cytotoxic effect of five subfractions on normal mouse liver cells (FL83B) was assessed by the MTT assay, and the results are presented in Figure S1. The subfractions did not significantly affect the cell proliferation of normal mouse liver cells FL83B with the highest concentration tested. Figure S1: cell viability of subfractions from black rice leaf on normal mouse liver cells (FL83B) were determined using MTT assay. Cells were treated with 0–400 *μ*g/mL of subfractions or 25 *μ*g/mL of 5-FU for 72 h. Table S1 Correlation coefficients between phytochemical content, antioxidant activity, and antiproliferative activity of subfractions derived from black rice leaf.
