# Supplementary material for: Antioxidant Activity and Inhibitory Effects of Black Rice Leaf on the Proliferation of Human Carcinoma Cells
Source: Biomed Res Int. 2022 Jun 11;2022:7270782. doi: 10.1155/2022/7270782 (PMC9206558; doi:10.1155/2022/7270782)
Supplement: Supplementary Materials — The cytotoxic effect of five subfractions on normal mouse liver cells (FL83B) was assessed by the MTT assay, and the results are presented in Figure S1. The subfractions did not significantly affect the cell proliferation of normal mouse liver cells FL83B with the highest concentration tested. Figure S1: cell viability of subfractions from black rice leaf on normal mouse liver cells (FL83B) were determined using MTT assay. Cells were treated with 0–400 μg/mL of subfractions or 25 μg/mL of 5-FU for 72 h. Table S1 Correlation coefficients between phytochemical content, antioxidant activity, and antiproliferative activity of subfractions derived from black rice leaf. [file 7270782.f1.docx]

**Supplementary Material**

The cytotoxic effect of five subfractions on normal mouse liver cells (FL83B) was assessed by the MTT assay, and the results are presented in Figure S1. The subfractions did not significantly affect the cell proliferation of normal mouse liver cells FL83B with the highest concentration tested.

Figure S1. Cell viability of subfractions from black rice leaf on normal mouse liver cells (FL83B) were determined using MTT assay. Cells were treated with 0–400 μg/mL of subfractions or 25 μg/mL of 5-FU for 72 h.

Table S1. Correlation coefficients between phytochemical content, antioxidant activity, and antiproliferative activity of subfractions derived from black rice leaf.

|  | **Phytochemical content** | | **Antioxidant activity** | | | **Antiproliferative activity** | | |
| --- | --- | --- | --- | --- | --- | --- | --- | --- |
|  | **TPC** | **TFC** | **DPPH** | **ABTS** | **Reducing power** | **HepG2** | **MCF-7** | **Caco-2** |
| **TPC** | 1 | 0.711^**^ | 0.950^**^ | 0.934^**^ | 0.957^**^ | 0.590^*^ | 0.952^**^ | 0.775^**^ |
| **TFC** |  | 1 | 0.578^*^ | 0.492 | 0.590^*^ | 0.688^**^ | 0.800^**^ | 0.980^**^ |
| **DPPH** |  |  | 1 | 0.989^**^ | 0.996^**^ | 0.330 | 0.817^**^ | 0.625^*^ |
| **ABTS** |  |  |  | 1 | 0.980^**^ | 0.277 | 0.792^**^ | 0.549^*^ |
| **Reducing power** |  |  |  |  | 1 | 0.378 | 0.833^**^ | 0.623^*^ |
| **HepG2** |  |  |  |  |  | 1 | 0.779^**^ | 0.753^**^ |
| **MCF-7** |  |  |  |  |  |  | 1 | 0.878^**^ |
| **Caco-2** |  |  |  |  |  |  |  | 1 |

TPC, total phenolic content; TFC, total flavonoid content; Statistical significance: * *p* < 0.05, ** *p* < 0.01.
